# Causes and remedies for low research productivity among postgraduate scholars and early career researchers on non-communicable diseases in Nigeria

**DOI:** 10.1186/s13104-019-4458-y

**Published:** 2019-07-15

**Authors:** Mojisola Morenike Oluwasanu, Ntekim Atara, Williams Balogun, Olutosin Awolude, Olayinka Kotila, Toyin Aniagwu, Prisca Adejumo, Omobolanle Olaronke Oyedele, Millicent Ogun, Ganiyu Arinola, Chinedum Peace Babalola, Christopher Sola Olopade, Olufunmilayo I. Olopade, Oladosu Ojengbede

**Affiliations:** 1 Department of Health Promotion and Education, African Regional Health Education Centre, Faculty of Public Health, College of Medicine, University of Ibadan, Ibadan, Nigeria; 2 Department of Radiation Oncology, Faculty of Clinical Sciences, College of Medicine/University College Hospital, University of Ibadan, Ibadan, Nigeria; 3 Department of Medicine, Faculty of Clinical Sciences, College of Medicine, University of Ibadan/University College Hospital, Ibadan, Nigeria; 4 Department of Obstetrics and Gynecology, Faculty of Clinical Sciences, College of Medicine, University of Ibadan/University College Hospital, Ibadan, Nigeria; 5 Department of Pharmaceutical Chemistry, Faculty of Pharmacy, University of Ibadan, Ibadan, Nigeria; 6 Centre for Drug Discovery Development and Production (CDDDP), Faculty of Pharmacy, University of Ibadan, Ibadan, Nigeria; 7 School of Occupational Health Nursing, University College Hospital, Ibadan, Nigeria; 8 Department of Nursing, Faculty of Clinical Sciences, College of Medicine, University of Ibadan, Ibadan, Nigeria; 9 Center for Population and Reproductive Health, College of Medicine, University of Ibadan, Ibadan, Nigeria; 10 Department of Chemical Pathology, Faculty of Clinical Sciences, College of Medicine, University College Hospital, University of Ibadan, Ibadan, Nigeria; 11 Center for Global Health, University of Chicago, Chicago, USA

**Keywords:** Non-communicable diseases, Early career researchers, Postgraduate students, Research productivity

## Abstract

**Objective:**

The aim of the descriptive, cross sectional, questionnaire-based study reported here was to explore the causes of low productivity in non-communicable diseases research among postgraduate scholars and early career researchers in Nigeria and identify measures that could facilitate increased research output.

**Results:**

The 89 respondents were masters-level, doctoral scholars and resident doctors who attended a workshop. Majorities of the respondents (over 70%) either agreed or strongly agreed that factors contributing to poor non-communicable diseases research productivity include a dearth of in-country researchers with specialized skills, inability of Nigerian researchers to work in multidisciplinary teams, poor funding for health research, sub-optimal infrastructural facilities, and limited use of research findings by policy makers. Almost all the respondents (over 90%) agreed that potential strategies to facilitate non-communicable diseases research output would include increased funding for research, institutionalization of a sustainable, structured capacity building program for early career researchers, establishment of Regional Centers for Research Excellence, and increased use of research evidence to guide government policy actions and programs.

**Electronic supplementary material:**

The online version of this article (10.1186/s13104-019-4458-y) contains supplementary material, which is available to authorized users.

## Introduction

Non-communicable diseases (NCDs) are the leading cause of deaths globally, accounting for 71% of all-cause mortality and over three quarters of these occurred in low and middle income countries [1]. One of the six prioritized objectives for countries in the 2013–2020 WHO Global Action Plan for NCDs is “to promote and support national capacity for high-quality research and development for the prevention and control of NCDs” [2].

Research is crucial to inform strategies that will ensure African countries are poised to achieve the targets for the NCDs-related Sustainable Development Goals (SDGs). Researchers at academic institutions should be at the fore in generating evidence to influence national health policies, improved service delivery and attainment of the goals but they are hindered by several factors [3, 4]. Achieving these goals requires critical analysis of factors that promote or hamper the NCDs research productivity of postgraduate students and early career researchers in Nigeria. Such analysis will guide development and implementation of appropriate multi-level interventions to strengthen research capacity in Nigeria and catalyze research productivity. Early career researchers play pivotal roles in the creation of new knowledge. Therefore, identification of factors influencing these scholars’ research productivity is pertinent to development of innovative research and discoveries that can lead to control of NCDs. Thus, a thorough understanding of the challenges early career researchers experience is both timely and pertinent for countries in Africa and globally [5].

The aim of this study was to identify and describe the factors that promote or hamper NCDs research among postgraduate students and early career researchers in Nigeria. The results will contribute to formulating plans to improve the quality and increase the quantity of NCDs research in the country.

## Main text

### Methods

This was a descriptive, cross sectional study. The protocol for the study was approved by the University of Ibadan, University College Hospital Ethical Review Committee. All respondents provided written consent to participate in the study. Participants were early career researchers and postgraduate students (masters and doctoral students, resident doctors, post-doctoral scholars and junior faculty members) who participated in a workshop organized by the Center for Population and Reproductive Health, College of Medicine, University of Ibadan, Nigeria in collaboration with the Center for Global Health, University of Chicago, USA. The workshop was held at the University of Ibadan, Center for Sustainable Development, Nigeria on the 22nd of March 2018. This workshop is one of the series of activities supported by a planning grant from the Fogarty International Center, National Institute of Health titled, “Addressing NCDs in Nigeria through Enhanced International Partnership and Interdisciplinary Research Training”. The goal of the effort was to assess needs and to plan for the development of a research training program to be funded by an NIH D43 grant. The encompassing project is to sustainably strengthen the research capacity of Nigeria academic and health institutions and to train in-country experts to conduct research on NCDs.

There was a university-wide advertisement of the workshop through the university bulletin coupled with dissemination through posters in strategic places. Applicants completed an application form and submitted the abstract of a completed or proposed NCDs research study which were the criteria for invitation and participation in the workshop. The participants completed a self-administered questionnaire prior the commencement of the workshop. Three members of the research team searched the literature based on the objective of the study and relevant contents were used to develop the questionnaire. Six people including nonprofessionals were appointed to review the questionnaire. To establish content validity, a panel of four seasoned NCDs researchers reviewed the questionnaire and their inputs were incorporated. The questionnaire was pre-tested among 10 post-graduate scholars and the reliability coefficient (alpha) was 0.8. The questionnaire had sections on the socio-demographic characteristics of respondents, the individual, organizational and policy-level factors that influence NCD research in Nigeria and strategies for enhancing NCD research output. There were 28 items to be rated on a 5-point Likert scale. These comprised 9 items about individual and organizational level factors affecting NCDs research output, 10 items about policy level factors, and 9 items about strategies to enhance research productivity. SPSS version 21 was used for data entry and descriptive statistical analysis.

### Results

A total of 89 participants drawn from seven research, academic, and health institutions in Nigeria took part in the study. Over half (55.1%) of the respondents were female and three-quarters (75.3%) were aged 40 years or less. More than a third (36%) had Master of Science degrees while 23.6% were doctoral students or resident doctors. Details in Additional file 1.

#### Individual- and organization-level factors contributing to low NCD research productivity

Table 1 summarizes participant responses with regard to the individual- and organization-level factors that contribute to low NCDs research productivity. Over 80% of the respondents agreed that this low productivity was due to the inability of Nigerian researchers to work in multidisciplinary collaborations (40.4% strongly agreed; 44.9% agreed). In a similar vein, a majority of the respondents agreed that a dearth of in-country researchers with specialized skills was a major impediment to research productivity (36% strongly agreed; 49.4% agreed). Further, about one-sixth (13.5%) strongly agreed, while 40.4% agreed, that lack of interest in NCDs compared to infectious diseases on the part of Nigerian researchers, contributes to the fairly low NCDs research output. Majority of respondents agreed that the paucity of quality scientific meetings (15.7% strongly agreed; 44.9% agreed) and poor remuneration of scientists (23.6% strongly agree; 49.4% agreed) were factors as well.

**Table 1:** Individual and organization-level factors contributing to low NCD research productivity in Nigeria (N = 89)

Variables	Strongly agree	Agree	Neutral	Disagree	Strongly disagree
Strong reliance on traditional medicine	8 (9.0)	34 (38.2)	18 (20.2)	24 (27.0)	5 (5.6)
Preponderance of faith-based healing	12 (13.5)	38 (42.7)	19 (21.3)	18 (20.2)	2 (2.2)
Low level of NCDs morbidity and mortality	1 (1.1)	2 (2.2)	4 (4.5)	31 (34.8)	51 (57.3)
Low level of risk factors for NCDs in Nigeria	1 (1.1)	2 (2.2)	4 (4.5)	31 (34.8)	51 (57.3)
Lack of in-country researchers	32 (36.0)	44 (49.4)	7 (7.9)	5 (5.6)	1 (1.1)
Poor remuneration of scientists in the country	21 (23.6)	44 (49.4)	14 (15.7)	9 (10.3)	1 (1.1)
Research on NCDs are more difficult than other health aspects	4 (4.5)	13 (14.6)	13 (14.6)	42 (47.2)	17 (19.1)
Lack of interest in NCD research by Nigerian scientists	12 (13.5)	36 (40.4)	18 (20.2)	17 (19.1)	6 (6.7)
Outdated science education curricula	17 (19.1)	37 (41.6)	11 (12.3)	18 (20.2)	6 (6.7)
Low number of high impact science journals in Nigeria	12 (13.5)	25 (28.1)	15 (16.8)	27 (30.3)	10 (11.2)
Paucity of quality scientific meetings in Nigeria	14 (15.7)	40 (44.9)	16 (17.9)	15 (16.9)	4 (4.5)
Nigerian scientists work more in silos	36 (40.4)	40 (44.9)	43 (4.5)	6 (6.7)	3 (3.4)

#### Policy-level factors contributing to low NCD research output

Table 2 summarizes policy-level factors limiting NCDs research output. According to the respondents, these included poor funding for health research (58.5% strongly agreed; 28.1% agreed) and limited use of research findings by policy makers (61.8% strongly agreed; 32.6% agreed). Other factors identified were the poor funding support from corporations and sub-optimal facilities infrastructure facilities (71.9% and 71.9%, respectively, strongly agreed or agreed).

**Table 2:** Policy-level factors contributing to low NCD research output in Nigeria (N = 89)

Variables	Strongly agree	Agree	Neutral	Disagree	Strongly disagree
Underfunding of health research	52 (58.5)	25 (28.1)	8 (9.0)	2 (2.2)	2 (2.2)
Poor support from corporate entities for research sponsorship	28 (31.5)	36 (40.4)	16 (18.0)	4 (4.5)	5 (5.6)
Insecurity in the country	13 (14.6)	33 (37.1)	19 (21.3)	15 (16.9)	9 (10.1)
Brain drain	25 (28.1)	23 (25.8)	16 (18.0)	19 (21.3)	6 (6.7)
Lack of infrastructural facilities	28 (31.5)	36 (40.4)	8 (8.1)	13 (14.6)	4 (4.5)
Lack of recognition of scientists in the country	13 (14.6)	44 (49.4)	9 (10.1)	14 (15.7)	7 (7.9)
High level of unemployment among Nigerian scientists	16 (18.0)	39 (43.8)	13 (14.6)	18 (20.2)	3 (3.4)
Low number of research institutions	15 (16.9)	30 (33.7)	10 (11.2)	25 (28.1)	9 (10.1)
Limited use of research findings by policy makers	55 (61.8)	29 (32.6)	3 (3.3)	1 (1.1)	1 (1.1)

#### Strategies for increasing NCD research output in Nigeria

Table 3 summarizes responses concerning strategies that have potential to enhance NCDs research productivity in Nigeria. Almost all of the respondents strongly agreed that the establishment of a Regional Center for Research Excellence has the potential to catalyze NCDs research and generate new knowledge (80.9%). Other factors include establishment of a statutory percentage allocation of special funds such as the Nigerian Tertiary Education Trust Fund (TETFund) and the National Health Insurance Scheme (NHIS) toward NCD-based research (93.3% strongly agreed or agreed), and governmental commitments to ensure NCD research outputs are translated into policy actions (80.9% strongly agreed; 15.7% agreed). In a similar vein, the respondents acknowledged that the institutionalization of a sustainable, structured, capacity building program for early career researchers has the potential to increase research output in Nigeria (82% strongly agreed).

**Table 3:** Strategies for enhancing NCD research output in Nigeria (N = 89)

Variables	Strongly agree	Agree	Neutral	Disagree	Strongly disagree
Establishment of Regional Centers for Research Excellence	72 (80.9)	13 (14.6)	3 (3.3)	(0)	1 (1.1)
Ease of acquisition of accurate and sequential data on NCDs	63 (70.8)	22 (24.7)	0 (0.0)	3 (3.3)	1 (1.1)
Introduction of electronic medical records to all tiers of the health sector	58 (65.2)	26 (29.2)	4 (4.5)	1 (1.1)	0 (0.0)
Inclusion of NCDs in global funding	71 (79.8)	16 (18.0)	0 (0.0)	2 (2.2)	0 (0.0)
Establishment of effective NCD department in the national and state ministries	57 (64.0)	26 (29.2)	5 (5.6)	0 (0.0)	1 (1.1)
Setting up a national population cancer registry	63 (70.8)	20 (22.5)	3 (3.4)	1 (1.1)	0 (0.0)
Establish a statutory percentage allocation of TETFund and NHIS funds toward NCDs research	55 (61.8)	28 (31.5)	5 (5.6)	1 (1.1)	0 (0.0)
Governmental commitments to ensure NCD research outputs influences policy formation	72 (80.9)	14 (15.7)	2 (2.2)	0 (0.0)	1 (1.1)
Structured capacity building in cutting-edge research techniques for early career researchers	73 (82.0)	13 (14.6)	1 (1.1)	2 (2.2)	(0)

### Discussion

There is a global call to improve investment in NCDs research. The Nigerian response, however, is impeded by multi-faceted challenges ranging from weak research capacity to poor government and donor funding and limited use of findings to drive health care policy. Similar to observations from a qualitative study conducted among early-career researchers from 10 African countries who attended a workshop in Malawi [6], this study revealed that limited research capacity, especially among early career researchers, remains an impediment to the conduct of innovative, transformative NCDs research that would align closely with national and regional priorities. This underscores the urgent need for investments in the development of NCD research capability and institutional infrastructure in low-income and middle-income countries as a strategic option for catalyzing innovations critical for NCDs prevention, diagnosis, treatment [7, 8] and the attainment of the SDGs.

This study revealed the limited opportunities for training, conferences and structured capacity building programs for early career researchers in Nigeria similar to findings among junior and mid-level medical faculty and researchers in Uganda [9] and Pakistan [10]. Nigeria has fostered development of a critical mass of NCDs early career researchers. However, these professionals need to be nurtured and mentored in order to compete internationally and thus diminish the disparities in research output between economically developed countries and LMICs. Indeed, there is a compelling need to nurture and mentor the next generation of NCDs researchers as a means of facilitating equitable research development and bridging the research divide [6, 9]. This support can be provided through institution-led, structured, career development programs and other opportunities, such as short- and long-term courses, mentoring and exchange programs, and in-country conferences and symposia.

The development of multidisciplinary teams with complementary expertise is central to NCDs research programs, as these have diverse components and the issues are nuanced and complex. Similar to findings from Uganda [9], Nigerian NCDs researchers see a need to work more in teams, so as to synergize their efforts. It is time to go beyond just putting NCDs researchers in Nigeria together in the workplace. They must be trained to work together [11].

Another worrisome challenge is the limited use of available research evidence to guide government policy actions and programs which aligns with findings from Malawi [6] and Turkey [12]. This could be due to the poor dissemination of research findings, making them unavailable for translation into policy and practice. It could also be due to the non-alignment of research studies with national/regional priorities [13]. To address such challenges, the Nigerian government needs to invest in and strengthen the national health research systems. Such actions will better enable research professionals to conceptualize, generate, and disseminate high-quality research evidence relevant to driving policies. In tandem, Nigerian researchers need to be responsive to national research needs and priorities.

Research leadership that promotes conceptualization, conduct and dissemination of NCDs research by Africans is central for tackling the development needs of the continent. Unfortunately, compared to other regions of the world, Africa has the lowest number of centers of research excellence that can generate ideas to tackle the continent’s myriad health and socioeconomic challenges [14]. Aligned with this observation, the respondents in this study agreed that establishment of Regional Centers for Research Excellence will be an effective approach to reducing the burden of NCDs in Nigeria and other LMICs. Regional centers can be expected, as well, to catalyze in-country research institutions to provide capacity for locally driven research efforts specific to the country’s settings [15].

Globally, insufficient funding remains a critical challenge for NCDs research. In response, the WHO constituted a Global Coordination Mechanism (GCM) to examine the current research financing landscape and explore sources of funding available to support domestic NCD research [16]. The group identified three key financing sources: domestic, overseas, and innovative and countries are expected to blend innovative and traditional funding sources. This GCM recommendation aligns with the questionnaire item that there be a statutory percentage allocation of TETFund and NHIS funding toward NCD-based research, with which a majority of respondents agreed. Other innovative funding mechanisms, such as the taxation of tobacco products, can be adopted to fund NCDs research in Nigeria [17].

NCD research in Nigeria faces several challenges at the individual, institutional and policy levels. Stimulating NCDs research will require timely and collaborative action from researchers, institutions and government. Priority actions include institutionally-led, structured career development and mentoring programs for early career researchers, establishment of Regional Centers for Research Excellence, translation of research evidence to policy actions and improved financing and investment in NCDs research by government and donor organizations. This ultimately will contribute to an improved NCDs research environment in Nigeria, making possible the prevention of NCDs and better care for individuals affected.

## Limitation

This was a convenience sample and respondents were not chosen according to a specific sampling strategy that had the potential to capture a broader spectrum of views. Therefore, the findings may not be representative of all postgraduate students and early career NCDs researcher in Nigeria. Nonetheless, the views expressed by the respondents are relevant to pressing issues in Nigeria and the findings form a good basis to address the challenges militating against the conduct of NCDs research in Nigeria.

## Additional file

**Additional file 1.** Socio-demographic characteristics of the respondents.

## Data Availability

The dataset is available upon request to the corresponding author. This can only be used for non-commercial purposes while maintaining participants’ confidentiality.

## Abbreviations

GCM: Global Coordination Mechanism
NCDs: non-communicable diseases
NHIS: National Health Insurance Scheme
RCRE: Regional Centre for Research Excellence
SDGs: Sustainable Development Goals
TETFund: Nigerian Tertiary Education Trust Fund
