# Supplementary material for: Causes and remedies for low research productivity among postgraduate scholars and early career researchers on non-communicable diseases in Nigeria
Source: BMC Res Notes. 2019 Jul 15;12:403. doi: 10.1186/s13104-019-4458-y (PMC6628473; doi:10.1186/s13104-019-4458-y)
Supplement: Supplementary file 1 — Additional file 1. Socio-demographic characteristics of the respondents. [file 13104_2019_4458_MOESM1_ESM.docx]

**Socio-demographic Characteristics of Respondents (N=89)**

| **Variable** | **Frequency** | | | **%** | |
| --- | --- | --- | --- | --- | --- |
| **Age** | | | | | |
| ≤40 years  ≥41 years | | 67  22 | | | 75.3  24.7 |
|  | |  | | |  |
| **Mean age** 36.2±8.9  **Sex** | | | | | |
| Male | | 40 | 44.9 | | |
| Female | | 49 | 55.1 | | |
|  | |  |  | | |
| **Institution**  University of Ibadan & University 79 89.8  College Hospital  University of Lagos 1 1.1  Ladoke Akintola University 1 1.1  Ekiti State University 1 1.1  University of Abuja 1 1.1  Obafemi Awolowo University 5 5.8  **Educational level/Highest Qualification**  Bachelors 9 10.1  Masters/Masters Public Health (*in view)* 13 14.6  Masters/Masters Public Health (*completed)* 32 36.0  Doctoral/Residency Program (in view) 21 23.6  Doctoral *(completed)* 13 14.6  Fellow West African Post Graduate Medical  College 1 1.1 | | | | | |
|  |  | | |  | |
